# Effects of inclusion of black soldier fly larvae on growth performance, relative organ weight, and meat quality of broiler chickens

**DOI:** 10.1016/j.psj.2025.105208

**Published:** 2025-04-23

**Authors:** H.N. Lee, K.H. Yum, G.L. Yeom, Y.B. Kim, J.Y. Park, S. Park, G. Park, Y. Choi, J. Choi, J.H. Kim

**Affiliations:** a Department of Animal Science, Chungbuk National University, Cheongju 28644, Republic of Korea; b Research Center, Orge CO., LTD., Jecheon 27157, Republic of Korea

**Keywords:** Black soldier fly larvae, Breast meat, Broiler chicken, Fatty acid, Growth performance

## Abstract

The objective of this experiment was to investigates effects of inclusion of black soldier fly larvae (BSFL) on growth performance, relative organ weight, and meat quality of broiler chickens. A total of 180 1-d-old broiler chickens were randomly allotted to 1 of 3 dietary treatments with 5 replicates. Each replicate consisted of 12 birds. Experimental diets were formulated to contain full-fat BSFL at inclusion levels of 0 %, 1 %, and 2 %. These diets were provided on an ad libitum basis for 5 wk. Results indicated that birds fed diet containing 1 % BSFL had greater (*P* < 0.05) feed efficiency than those fed diets containing 2 % BSFL. However, BW gain and feed intake of broiler chickens were not affected by increasing inclusion levels of BSFL in diets. Increasing inclusion levels of BSFL in diets showed a quadratic relationship (*P* < 0.05*)* with relative thymus weight. For meat color, values for redness (a*) and yellowness (b*) were decreased (linear, *P* < 0.05) as BSFL inclusion level of diets increased. Thiobarbituric acid reactive substance decreased (quadratic, *P* < 0.05) with increasing inclusion levels of BSFL in diets. Melanin concentrations in breast meat and liver characteristics were not affected by increasing inclusion levels of BSFL in diets. Increasing concentrations of BSFL in diets increased myristic acid concentrations (linear, *P* < 0.01) and eicosapentaenoic acid concentrations (linear, *P* < 0.05) in breast meat. These results suggest that dietary supplementation of BSFL has positive effects on immune organ weight, breast meat quality, and fatty acid compositions in breast meat. Therefore, BSFL can be used as a feed ingredient for broiler chickens. Also, we suggest that dietary supplementation of 1 % BSFL improves the performance and health of broiler chickens.

## Introduction

Global population is anticipated to increase to about 9.2 billion people by the yr 2050 (Ndotono et al., 2022). Meat consumption is steadily increasing as the population grows. The world population is expected to increase by 11 % between 2021 and 2030, and global meat consumption is expected to increase by 14 % in 2030 compared to that about 10 yr ago (Farchi et al., 2017; Godfray et al., 2018; OECD/FAO, 2023). With the increasing human population, demand for meat consumption is also increasing, subsequently increasing demand for protein sources (Affedzie-obresi et al., 2020). Additionally, increasing demand for protein sources has led to competition for resources (Schiavone et al., 2017a). In addition, food and feed supplies are unstable due to conflict, natural climate cycles, anthropogenic climate changes, livestock production, and health crises (GNAFC, 2022).

Protein is an essential feed ingredient and one of the most expensive components required for livestock feed (Beski et al., 2015). However, prices of protein ingredients such as soybean and fish meals are increasing (Kim et al., 2021a) due to several factors, including a lack of land for production, global cost fluctuations, human consumption of grains, and other constraints (Onsongo et al., 2018). Accordingly, insects are attracting attention as an alternative new source of protein (Kim et al., 2021a). Among various insects, black soldier fly larvae (*Hermetia illucens*; BSFL) have emerged as one of the promising insects that can be used as feed components (Heita et al., 2023; Khan et al., 2018). The reason is because BSFL can rapidly consume large quantities of organic waste such as spoiled feed, food, and manure. Moreover, BSFL can convert low quality of organic waste into high quality of proteins and fat (Dörper et al., 2021). Thus, treating organic waste with BSFL is a promising technology that can reduce and recycle food wastes responsible for unsanitary conditions encountered in urban areas into useful products (Dzepe et al., 2021).

The BSFL contains high levels of protein, fat, amino acids, and fatty acids that are favorable for poultry nutrition (Veldkamp and Bosch, 2015). In particular, BSFL contains high levels of saturated fatty acids (SFAs), including lauric acid and myristic acid. (Benzertiha et al., 2020; Li et al., 2016; Ushakova et al., 2016). Medium-chain fatty acids (MCFAs), such as lauric acid, have been reported to exert antibacterial effects in broiler chickens (Pappula et al., 2021). In addition, lauric and myristic acids have been shown to improve growth performance and meat quality. (Zeitz et al., 2015). Previous studies have shown that dietary supplementation with 3 % to 20 % BSFL had no adverse effects on broiler chickens (Choi et al., 2013; Herawati and Permata, 2023). However, limited research has been conducted on the effects of including full-fat BSFL powder at levels below 3 % in broiler diets.

For this reason, increasing concentrations of dietary BSFL are expected to improve growth performance, meat quality, and antibacterial effects. However, data regarding the use of full-fat BSFL powder in broiler diets remain limited. Moreover, no studies have investigated the effects of full-fat BSFL powder at inclusion levels below 3 % in broiler diets. Therefore, the objective of this experiment was to investigate the effects of dietary full-fat BSFL supplementation on the growth performance and health of broiler chickens. Specifically, this study examined the effects of two inclusion levels (i.e., 1 % and 2 %) of full-fat BSFL on growth performance, relative organ weight, and meat quality in broiler chickens.

## Materials and methods

### Animal ethics statement

All experimental procedures were reviewed and approved by the Institutional Animal Care and Use Committee at Chungbuk National University (CBNUA-2107-23-01).

### Animals, diets, and experimental design

A total of one hundred eighty 1-d-old Ross 308 broiler chicks were obtained from a local commercial hatchery (Dongsan Hatchery, Cheonan, Republic of Korea) and transferred in an environmentally controlled room. All birds were allotted to each floor pen with a similar average BW (BW ± SD = 36.33 ± 0.1 g) among pens. All birds were selected and allotted to 1 of 3 dietary treatments with 5 replicates, each replicate consisting of 12 birds in a completely randomized design. A total of 180 broiler chicks (90 males and 90 females) were allocated to the experimental treatments, with each replicate comprising 6 males and 6 females. All diets were formulated to meet or exceed the Ross 308 broiler nutrition specifications (Aviagen, 2022) for energy and nutrients in broiler chickens (Table 1). Energy and nutrient concentrations of all experimental diets were analyzed (Table 2). Experimental diets were formulated to contain full-fat BSFL at inclusion levels of 0 %, 1 %, and 2 %. Assumed AME_n_ value (5,436.22 kcal/kg) for BSFL was calculated from the prediction equation of corn co-products in broiler chickens (Rochell et al*.,* 2011). Amino acids, CP, calcium, and phosphorus concentrations in BSFL were determined based on analyzed nutrient compositions of BSFL (Table 2). The BSFL were provided by Ara Insect (Haman-gun, Gyeongsangnamdo, Republic of Korea). Nutrient compositions of full-fat BSFL were analyzed and presented in Table 2. During the experiment, experimental diets and water were fed to birds on an ad libitum for 5 wk. Birds were exposed to a lighting schedule of 24 h light. During the first 3 days of the experiment, the temperature and relative humidity were maintained at 30°*C* ± 0.5 and 67.7 % ± 2.62, respectively. And then gradually decreased to 21°C at the end of the experiment as recommended by Ross 308 broiler pocket guide (Aviagen, 2022). The BW gain (BWG) and feed intake (FI) were recorded at the conclusion of the experiment. Feed efficiency (FE) was calculated by dividing BWG with FI. Additionally, FI was adjusted with the number and living days of dead birds.

**Table 1:** Composition and nutrient content of the experimental diets (as-fed basis).

	Inclusion levels of BSFL in diets (0 to 3 wk), %			Inclusion levels of BSFL in diets (4 to 5 wk), %		
Items	0	1	2	0	1	2
Ingredient, %						
Corn	52.01	53.58	53.89	57.15	58.57	59.68
Soybean meal, 46 % CP	30.14	30.51	33.03	28.00	28.70	29.87
Corn gluten meal	7.10	5.97	3.47	4.00	2.65	1.01
Tallow	3.71	3.00	2.92	4.85	4.21	3.73
Celite	2.00	1.00	0.00	2.00	1.00	0.00
BSFL	0.00	1.00	2.00	0.00	1.00	2.00
Salt	0.30	0.30	0.30	0.30	0.30	0.30
MDCP	1.74	1.72	1.66	1.32	1.29	1.24
Limestone	1.22	1.18	1.11	0.87	0.82	0.78
98.5 % Met	0.38	0.39	0.41	0.34	0.35	0.37
55 % Lys H_2_SO_4_	0.69	0.64	0.50	0.50	0.44	0.35
98.5 % Thr	0.16	0.16	0.16	0.12	0.12	0.12
50 % choline	0.10	0.10	0.10	0.10	0.10	0.10
NaHCO_3_	0.15	0.15	0.15	0.15	0.15	0.15
Vitamin premix^1^	0.15	0.15	0.15	0.15	0.15	0.15
Mineral premix^2^	0.15	0.15	0.15	0.15	0.15	0.15
Total	100.00	100.00	100.00	100.00	100.00	100.00
Calculated energy and nutrient content^3^						
AME_n_, kcal/kg	3,016	3,016	3,016	3,100	3,100	3,100
CP, %	22.32	22.32	22.32	19.66	19.66	19.66
Digestible Lys, %	1.26	1.26	1.25	1.09	1.09	1.08
Digestible Met + Cys, %	0.98	0.98	1.00	0.87	0.88	0.89
Digestible Met, %	0.68	0.69	0.70	0.60	0.61	0.62
Digestible Thr, %	0.84	0.85	0.87	0.73	0.74	0.75
Digestible Trp, %	0.21	0.22	0.23	0.19	0.20	0.21
Total calcium, %	0.86	0.86	0.86	0.66	0.66	0.66
Available phosphorus, %	0.47	0.47	0.47	0.37	0.37	0.37

Abbreviation: BSFL = black soldier fly larvae; MDCP = monodicalciumphosphate.

1 Provided per kilogram of the complete diet: vitamin A (from vitamin A acetate), 13,000 IU; vitamin D_3_, 5,000 IU; vitamin E (from DL-α-tocopheryl acetate), 80 IU; vitamin K_3_, 4 mg; vitamin B_1_, 4 mg; vitamin B_2_, 10 mg; vitamin B_6_, 6 mg; vitamin B_12_, 20 µg; calcium pantothenate, 20 mg; folic acid, 2 mg; biotin, 200 µg; niacin, 60 mg.

2 Provided per kilogram of the complete diet: Zn (as ZnO), 100 mg; Mn (as MnSO_2_·H_2_O), 120 mg; Fe (as FeSO_4_·7H_2_O), 60 mg; Cu (as CuSO_4_·5H_2_O), 16 mg; Co (as CoCO_3_), 1,000 µg; I (as Ca (IO_3_)_2_·H_2_O), 1.25 mg; Se (as Na_2_SeO_3_), 300 µg.

3 Calculated values from the Arbor Acres broiler nutrition specifications (Aviagen, 2022). Assumed AME_n_ value for BSFL (5436.22 kcal/kg) was calculated from the prediction equation of corn co-products in broiler chickens (Rochell et al., 2011).

**Table 2:** Analyzed energy and nutrient compositions of black soldier fly larvae (BSFL) used in this experiment (as-fed basis).

	Full-fat BSFL	Inclusion levels of BSFL in diets (0 to 3 wk), %			Inclusion levels of BSFL in diets (4 to 5 wk), %		
Items		0	1	2	0	1	2
Gross energy, kcal/kg	5,436	3,983	4,031	4,074	4,123	4,087	4,103
DM, %	96.69	89.93	89.73	89.75	89.55	89.41	89.18
Crude ash, %	16.00	8.18	6.66	6.12	6.20	6.06	5.01
CP, %	37.91	22.31	22.38	22.74	20.08	19.21	18.87
Ether extract, %	29.19	5.48	5.27	7.07	7.73	6.90	6.48
Calcium, %	4.80	0.88	0.83	0.85	0.67	0.65	0.71
Phosphorus, %	0.59	0.70	0.67	0.64	0.57	0.55	0.53
Melanin, ug/g	782	179	249	218	168	257	389
Essential amino acid, %							
Arg	1.83	1.08	1.14	1.32	1.06	1.03	1.01
His	1.15	0.50	0.52	0.55	0.49	0.44	0.46
Ile	1.60	0.81	0.84	0.91	0.78	0.69	0.72
Leu	2.59	2.22	2.11	2.09	1.90	1.59	1.59
Lys	2.22	1.25	1.39	1.43	1.14	1.10	1.05
Met	0.76	0.70	0.68	0.72	0.56	0.66	0.61
Phe	1.55	1.08	1.08	1.13	0.99	0.86	0.88
Thr	1.47	0.94	1.00	0.97	0.86	0.84	0.83
Trp	0.44	0.22	0.18	0.20	0.18	0.17	0.16
Val	2.37	0.94	0.95	1.02	0.88	0.80	0.83
Non-essential amino acid, %							
Ala	2.59	1.32	1.23	1.22	1.09	0.95	0.95
Asp	3.30	1.82	1.89	2.15	1.77	1.61	1.70
Cys	0.38	0.45	0.46	0.49	0.43	0.42	0.42
Glu	4.04	3.86	3.76	4.01	3.47	3.03	3.08
Gly	2.06	0.81	0.83	0.91	0.76	0.74	0.74
Pro	2.18	1.46	1.40	1.42	1.27	1.10	1.09
Ser	1.57	1.05	1.03	1.12	0.95	0.86	0.87
Tyr	1.99	0.63	0.59	0.70	0.48	0.54	0.44
Saturated fatty acid, g/100 g							
Capric acid (C6:0)	0.01	0.05	0.05	0.04	0.04	0.05	0.07
Caprylic acid (C8:0)	0.01	0.03	0.03	0.02	0.03	0.02	0.03
Capric acid (C10:0)	1.51	0.16	0.10	0.12	0.10	0.18	0.16
Lauric acid (C12:0)	35.75	0.44	1.71	3.29	0.15	1.42	2.60
Myristic acid (C14:0)	5.10	1.04	1.18	1.42	1.27	1.33	1.41
Pentadecanoic acid (C15:0)	0.12	0.10	0.08	0.10	0.11	0.11	0.10
Palmitic acid (C16:0)	14.05	18.72	18.34	18.49	20.44	19.34	18.92
Margaric acid (C17:0)	0.18	0.25	0.27	0.28	0.30	0.32	0.26
Stearic acid (C18:0)	2.80	7.88	7.53	7.47	9.61	8.19	7.88
Arachidic acid (C20:0)	0.04	0.00	0.00	0.00	0.00	0.00	0.00
Heneicosanoic acid (C21:0)	0.10	0.06	0.05	0.04	0.05	0.05	0.05
Behenic acid (C22:0)	0.05	0.24	0.27	0.22	0.28	0.25	0.24
Lignoceric acid (C24:0)	0.02	0.06	0.05	0.04	0.07	0.06	0.05
Unsaturated fatty acid, g/100 g							
Myristoleic acid (C14:1)	0.26	0.15	0.11	0.15	0.17	0.16	0.15
Pentadecanoic acid (C15:1)	0.08	0.05	0.03	0.03	0.04	0.03	0.03
Palmitoleic acid (C16:1)	3.12	1.68	1.72	1.84	2.04	2.03	1.89
Magaoleic acid (C17:1)	0.24	0.24	0.21	0.23	0.28	0.25	0.22
Oleic acid (C18:1 ω−9)	18.14	36.75	35.29	34.72	38.71	37.38	36.13
Linoleic acid (C18:2 ω−6)	14.31	29.21	30.12	28.57	23.76	26.17	26.30
γ-Linolenic acid (C18:3 ω−6)	0.16	0.33	0.20	0.31	0.27	0.31	0.31
Linolenic acid (C18:3 ω−3)	1.56	1.40	1.43	1.48	1.07	1.16	1.47
Eicosenoic acid (C20:1 ω−9)	0.57	0.69	0.66	0.64	0.77	0.68	1.15
Eicosadienoic acid (C20:2 ω−6)	0.03	0.02	0.01	0.02	0.02	0.01	0.03
Dihomoγ-Linolenic acid (C20:3 ω−6)	0.00	0.02	0.02	0.02	0.02	0.02	0.04
Eicosatrienoic acid (C20: 3 ω−3)	0.00	0.01	0.00	0.01	0.01	0.02	0.00
Arachidonic acid (C20:4 ω−6)	0.27	0.04	0.05	0.05	0.07	0.06	0.07
Eicosapentaenoic acid (C20:5 ω−3)	1.23	0.02	0.11	0.07	0.04	0.10	0.06
Erucic acid (C20:1 ω−9)	0.04	0.19	0.19	0.17	0.16	0.17	0.21
Docosadienoic acid (C22:2 ω−6)	0.04	0.09	0.12	0.09	0.07	0.08	0.10
Nervonic acid (C24:1 ω−9)	0.03	0.03	0.03	0.03	0.04	0.03	0.03
Docosahexaenoic acid (C22:6 ω−3)	0.18	0.04	0.04	0.05	0.03	0.04	0.05

### Sample collection

At the conclusion of experiment, 1 male broiler chicken with a BW close to the mean BW per pen was euthanized by CO_2_ asphyxiation and dissected immediately. The internal organs (i.e., breast, liver, spleen, kidney, bursa of Fabricius, and thymus) were excised and weighed to calculate relative organ weights, which were recorded as percentages of BW. Breast samples were sampled and stored at −20°C before analysis. Feather samples were obtained from the left wing and stored at −20°C until measurement of corticosterone. Fecal samples were collected at 50 g per pen and stored at −20°C before analysis.

### Breast meat quality assay

The pH was measured using a pH meter (Thermo Fisher Scientific, Waltham, MA) after adding 90 mL distilled water to 10 g sample and homogenizing at 10,000 rpm for 30 s using a homogenizer (Bihon Seiki, Ace, Osaka, Japan). Meat color was analyzed using a Spectro colorimeter (Konica Minolta, Tokyo, Japan) to obtain lightness (L*), redness (a*), and yellowness (b*) values. Cooking loss significantly affects the sensory qualities of meat (Martens et al., 1982). A high cooking loss indicates reduced juiciness of the meat (Kondjoyan et al., 2013). This factor is critical in determining the yield of cooking processes and holds significant importance in the meat industry (Macharáčková et al., 2021). Cooking loss was determined by heating each sample in a water bath at 70°C for 30 min and indicating weight difference before and after heating as a percentage. Drip loss adversely affects the meat industry due to its economic implications (Torres Filho et al., 2017). In addition, it leads to an unattractive meat appearance, resulting in lower consumer acceptance and, consequently, reduced sales (Otto et al., 2004). Drip loss was measured by vacuum packaging each sample in a refrigerator at 4°C for 24 h and indicating weight difference before and after storing as a percentage. Shear force is one of the methods used to objectively assess meat tenderness. Tenderness is a key component of meat quality that influences palatability (Jeremiah, 1982). Although tenderness is often evaluated subjectively by consumers, it can also be quantified using shear force analysis (Hwang et al., 2003; Karumendu et al., 2009). Shear force was measured using a Rheometer (Sun Scientific CO., LTD., Sakurashimmachi, Setagaya-ku, Tokyo, Japan) and presented as the maximum shear stress. Water holding capacity (WHC) is a crucial factor that influences both the economic value and overall quality of meat (Barbera, 2019). The WHC affects various meat quality attributes, including drip loss, weight loss, cooking shrinkage, juiciness, and tenderness (Gault, 1985; Lawrie, 1988). The WHC was determined using the method described by Laakkonen et al*.* (1970). Briefly, a 0.5 g sample was added to a filter tube and heated at 80°C for 20 min. After centrifuging at 2,000 rpm for 10 min, it was cooled for 10 min. Thiobarbituric acid reactive substance (TBARS) was determined using the method described by Witte et al. (1970). In short, 15 mL of 10 % perchloric acid (sample of 10 g diluted with 70 % perchloric acid, Samchun Chemicals, Pyeongtaek, Republic of Korea) and 20 mL distilled water were homogenized at 10,000 rpm for 30 s using a homogenizer. The homogenate was filtered using Whatman No 2. filter paper and 5 mL the filtrate was mixed with 5 mL of 2-TBA (Sigma Aldrich, Darmstadt, Germany). The mixture was cooled in a cold room for 16 h. After that, the absorbance was analyzed at 529 nm using a Spectrophotometer (Micro Digital CO., LTD., Seongnam, Republic of Korea). The CP, crude fat (CF), moisture, and crude ash concentrations in breast meat were determined according to the Association of Official Analytical Chemists (2007). The CP was analyzed using the Kjeldahl method, and the moisture concentration was measured by a 105°C air-oven method. The CF was measured using the Folch method (1957) and crude ash was analyzed using the 550°C ashing method with a muffle furnace. Sensory evaluation was conducted to assess the quality of cooked breast meat. Eight evaluators assessed a total of four factors (meat color, texture, and moisture exudation) using a 5-point scale (meat color: 1 point = pale, 5 point = dark; texture: 1 point = soft, 5 = firm; moisture exudation 1 point = low, 5 point = high).

The melanin concentrations were analyzed using breast meat that was dried using a drying oven at 70°C for 48 h and ground to a powder. A 50 mg sample of the dried sample was mixed with 10 % (v/v) dimethyl sulfoxide in 1 N NaOH. The mixture was heated using a heat block at 80°C for 30 min. It was vortexed at 10-min intervals. The sample was centrifuged at 17,000 rpm for 30 min, and the supernatant was collected for spectrophotometric analysis at 490 nm. The melanin content was measured through a standard curve of synthetic melanin (Sigma Aldrich, Darmstadt, Germany).

### Liver characteristics

Livers sampled at wk 5 were used to measure liver color immediately after sampling and to analyze subjective liver hemorrhagic and fatty liver scores. The color of a raw liver was measured using a color reader (Konica Minolta Optics Inc., Tokyo, Japan) to determine L*, a*, and b* values. Liver hemorrhagic and fatty liver color scores ranged from 0 to 5, with 0 indicating normal liver and 5 indicating large and massive hemorrhages (Choi et al., 2012; Diaz et al., 1999). Five evaluators assessed liver hemorrhagic and fatty liver color scores.

Antioxidant capacity of a liver such as malondialdehyde (MDA) and total antioxidant capacity (TAC) was analyzed using a commercially available Oxiselect^TM^ TBARS Assay kit (MDA Quantitation, STA-330, Cell Biolabs, Inc., San Diego, CA) and Oxiselect^TM^ Total Antioxidant Capacity Assay Kit (STA360, Cell Biolabs, Inc., San Diego, CA) according to each manufacturer’s protocol. Protein concentrations were measured using a Pierce BCA protein assay kit (Thermo Fisher Scientific, Rockford, IL). The relative concentrations of MDA and TAC to protein concentrations were calculated and expressed as µmol/mg, respectively.

### Fatty acid composition in breast meat

A 50 g sample and 150 mL chloroform:methanol (2:1) were homogenized at 2,500 rpm according to the method of Folch et al. (1957). After homogenization, lipids were extracted and moisture was removed using sodium sulfate anhydrous. The filtrate was concentrated at 50°C to 55°C. To 10 µL of the concentrated liquid, 1 mL of 0.5 N NaOH was added. The mixture was heated at 100°C for 20 min and then cooled for 30 min. After adding 2 mL BF_3_-methanol, the liquid was heated at 100°C for 20 min and then cooled for 30 min. Subsequently, 1 mL of heptane and 8 mL of NaCl were added. The supernatant was then collected and injected into a gas chromatograph (GC, Agilent Technologies Inc., Santa Clara, CA) for fatty acid analysis.

### Fecal index

Fecal index was analyzed for moisture and pH. Fecal moisture was analyzed according to the methods described by Ogunji et al., 1983 and Chavez et al. (2004). fecal samples were dried using a drying oven (Hanbeak Science, Bucheon, Republic of Korea) at 105°C for 24 h. Moisture was calculated as the percentage of water lost by the sample. The pH was measured using a pH meter (Hanna Instruments, Woonsocket, RI).

### Statistical analysis

All statistical analyses were performed as a completely randomized design using the PROC MIXED procedure of SAS (SAS Institute Inc., Cary, NC). Each replicate was considered as the experimental unit for all analyses. All data were checked for normal distribution and outliers were checked with the UNIVARIATE procedure of SAS (Steel et al*.,* 1997). The least significant difference test was conducted to calculate treatment means and the PDIFF option of SAS was used to separate means if the difference was significant. Orthogonal polynomial contrast tests were used to test linear and quadratic effects of increasing inclusion levels of BSFL in diets. Statistical significance level was set at *P <* 0.05.

## Results

### Growth Performance

Increasing inclusion levels of BSFL in diets did not significantly affect BW, BWG, or FI of broiler chickens (Table 3). Birds fed diets containing 0 %, 1 %, and 2 % BSFL did not show differences in BW, BWG, or FI at 0 to 3 wk or 4 to 5 wk. In the overall period, the FE of birds fed diet containing 1 % BSFL was greater (*P* < 0.05) than the FE of birds fed diets containing 2 % BSFL. However, BW, BWG, and FI were not influenced by increasing inclusion levels of BSFL in diets.

**Table 3:** Effects of increasing inclusion levels of black soldier fly larvae (BSFL) in diets on growth performance of broiler chickens.1.

	Inclusion levels of BSFL in diets, %				*P-*value		
Items	0	1	2	SEM	T	L	Q
0 to 3 wk							
BW, g	797	792	824	11.9	0.157	0.133	0.210
BWG, g	761	755	788	11.9	0.157	0.134	0.211
FI, g	1,193	1,159	1,279	33.1	0.064	0.093	0.082
FE, g/kg	640	653	618	15.2	0.296	0.323	0.225
4 to 5 wk							
BW, g	1,840	1,843	1,877	33.8	0.694	0.452	0.705
BWG, g	1,043	1,051	1,053	28.0	0.964	0.802	0.927
FI, g	1,715	1,713	1,807	46.5	0.298	0.187	0.413
FE, g/kg	608	614	583	11.3	0.152	0.133	0.201
0 to 5 wk							
BW, g	1,840	1,843	1,877	33.8	0.694	0.452	0.705
BWG, g	1,804	1,806	1,841	33.9	0.694	0.452	0.705
FI, g	2,909	2,872	3,086	63.2	0.073	0.070	0.131
FE, g/kg	621^a^^b^	629^a^	597^b^	8.2	0.044	0.064	0.068

Abbreviation: *T* = overall effects of treatments; *L* = linear effects of increasing inclusion levels of BSFL in diets; *Q* = quadratic effects of increasing inclusion levels of BSFL in diets; BWG = BW gain; FI = feed intake; FE = feed efficiency (BWG:FI).

1 Data are least squares means of 5 observations per treatment.

a,b Means within a variable with no common superscript differ significantly (*P* < 0.05).

### Relative organ weight

Relative thymus weight exhibited a quadratic relationship (*P* < 0.05*)* with increasing concentrations of BSFL in diets (Table 4)*.* However, birds fed diets containing 0 %, 1 %, and 2 % BSFL did not show differences in relative organ weights (liver, spleen, kidney, and bursa of Fabricius).

**Table 4:** Effects of increasing inclusion levels of black soldier fly larvae (BSFL) in diets on relative organ weight of broiler chickens.1.

	Inclusion levels of BSFL in diets, %				*P-*value		
Items^2^	0	1	2	SEM	T	L	Q
Liver, %	2.30	2.41	2.44	0.093	0.582	0.329	0.757
Spleen, %	0.13	0.08	0.10	0.016	0.133	0.204	0.110
Kidney, %	0.53	0.47	0.61	0.049	0.172	0.275	0.121
bursa of Fabricius, %	0.22	0.21	0.18	0.019	0.272	0.116	0.862
Thymus, %	0.36	0.46	0.34	0.037	0.092	0.632	0.035

Abbreviation: *T* = overall effects of treatments; *L* = linear effects of increasing inclusion levels of BSFL in diets; *Q* = quadratic effects of increasing inclusion levels of BSFL in diets.

1 Data are least squares means of 5 observations per treatment.

2 The relative organ weight was expressed as a percentage of BW.

### Breast meat quality

For meat color, values for a* and b* were decreased (linear, *P* < 0.05) when the BSFL inclusion level was increased in diets (Table 5). The shear force decreased (quadratic, *P* < 0.05) when the BSFL inclusion level was increased in diets. The TBARS (5 d) was decreased (quadratic, *P* < 0.05) with increasing concentrations of BSFL in diets. Increasing concentrations of BSFL in diets decreased (linear and quadratic, *P* < 0.01) meat color of sensory evaluation. The wetness was increased (linear, *P* < 0.01) as BSFL concentrations were increased in diets. Broiler chickens fed diets containing 2 % BSFL had less (*P* < 0.05) b* value for meat color than those fed diets containing 0 % and 1 % BSFL. The TBARS (5 d) value for birds fed diets containing 2 % BSFL was less (*P* < 0.05) than for those fed diets containing 1 % BSFL. Birds fed diets containing 1 % and 2 % BSFL had greater (*P* < 0.05) meat color in sensory evaluation than those fed diets containing 0 % BSFL. The wetness in sensory evaluation for 2 % BSFL was greater (*P* < 0.05) than those for 0 % and 1 % BSFL. Increasing inclusion levels of BSFL in diets had no significant effects on melanin concentrations in breast meat. In addition, melanin concentrations in breast meat showed no significant differences between basal diet and BSFL supplemented diets.

**Table 5:** Effects of increasing inclusion levels of black soldier fly larvae (BSFL) in diets on meat quality of broiler chickens.1.

		Inclusion levels of BSFL in diets, %				*P*-value		
Items		0	1	2	SEM	T	L	Q
Breast yield, %		22.10	22.70	22.70	0.580	0.696	0.483	0.645
pH		6.07	6.00	6.06	0.060	0.730	0.936	0.440
Meat color (CIE Lab value)	L*	46.76	48.86	47.07	0.922	0.135	0.607	0.226
	a*	4.38	3.95	2.89	0.312	0.131	0.006	0.420
	b*	19.07^a^	18.42^a^	15.97^b^	0.729	0.003	0.011	0.355
Cooking loss		12.59	14.15	16.67	1.813	0.311	0.138	0.832
Drip loss		1.70	1.56	1.78	0.260	0.834	0.824	0.584
Shear force		2,850	3,471	2,786	212.4	0.078	0.836	0.027
WHC		61.21	55.75	59.94	1.812	0.125	0.629	0.050
TBARS	0 d	0.11	0.11	0.13	0.016	0.766	0.489	0.850
	5 d	0.12^ab^	0.14^a^	0.10^b^	0.008	0.023	0.069	0.026
Crude ash, %		0.95	0.99	1.13	0.106	0.444	0.234	0.691
CP, %		22.69	22.66	22.67	0.302	0.998	0.973	0.962
Crude fat, %		2.00	2.29	1.74	0.140	0.134	0.325	0.082
Moisture, %		74.81	74.64	75.27	0.361	0.493	0.413	0.394
Melanin, mg/g		0.041	0.039	0.039	0.002	0.638	0.555	0.467
Sensory evaluation								
Color		2.74^b^	3.26^a^	3.19^a^	0.075	0.001	0.001	0.007
Texture		3.41	3.01	3.13	0.137	0.146	0.163	0.153
Wetness		2.68^c^	2.90^b^	3.13^a^	0.040	<0.001	<0.001	1.000

Abbreviation: *T* = overall effects of treatments; *L* = linear effects of increasing inclusion levels of BSFL in diets; *Q* = quadratic effects of increasing inclusion levels of BSFL in diets; L* = lightness; a* = redness; b* = yellowness (breast meat color); WHC = water holding capacity; TBARS = thiobarbituric acid reactive substance (malondialdehyde equivalents per g of meat sample).

1 Data are least squares means of 5 observations per treatment.

a-c Means within a variable with no common superscript differ significantly (*P* < 0.05).

### Liver characteristics

Liver color, liver hemorrhage, and fatty liver score were not affected by increasing inclusion levels of BSFL in diets (Table 6). Additional supplementation of BSFL in diets did not affect fatty liver incidence compared with a basal diet. Increasing inclusion levels of BSFL in diets had no significant effects on MDA or TAC in breast meat of broiler chickens. Additional supplementation of BSFL in diets did not affect antioxidant capacity in the liver.

**Table 6:** Effects of increasing inclusion levels of black soldier fly larvae (BSFL) in diets on liver characteristics of broiler chickens.1.

		Inclusion levels of BSFL in diets, %				*P-*value		
Items		0	1	2	SEM	T	L	Q
Liver color	L*	23.7	25.0	23.2	0.77	0.248	0.654	0.113
	a*	13.4	13.6	11.8	0.78	0.239	0.168	0.320
	b*	4.0	4.8	3.5	0.49	0.203	0.480	0.103
Liver hemorrhagic score		0.3	0.7	0.9	0.22	0.176	0.072	0.711
Fatty liver score		1.2	1.4	0.6	0.24	0.543	0.294	0.779
TAC, μM/mg protein		903	730	847	69.1	0.233	0.573	0.111
MDA, μM/mg protein		3.17	3.26	3.48	0.29	0.751	0.480	0.860

Abbreviation: *T* = overall effects of treatments; *L* = linear effects of increasing inclusion levels of BSFL in diets; *Q* = quadratic effects of increasing inclusion levels of BSFL in diets; L* = lightness; a* = redness; b* = yellowness (Liver color); TAC = total antioxidant capacity; MDA = malondialdehyde.

1 Data are least squares means of 5 observations per treatment.

### Fatty acid concentrations in breast meat

Increasing inclusion levels of BSFL in diets increased (linear, *P* < 0.01) myristic acid concentrations in breast meat (Table 7). Eicosapentaenoic acid (EPA) concentrations in breast meat were also increased (linear, *P* < 0.05) as BSFL concentrations of diets were increased. Broiler chickens fed diets containing 2 % BSFL had greater (*P* < 0.05) myristic acid concentrations in breast meat than those fed diets containing 0 % and 1 % BSFL. However, dietary supplementation of BSFL had no significant effects on fatty acids except for myristic acid and EPA.

**Table 7:** Effects of increasing inclusion levels of black soldier fly larvae (BSFL) in diets on fatty acid concentrations (% of total fatty acid) in muscle of broiler chickens.1.

	Inclusion levels of BSFL in diets, %				*P-*value		
Items	0	1	2	SEM	T	L	Q
Myristic acid (C14: 0)	1.07^c^	1.14^b^	1.31^a^	0.023	<0.001	<0.001	0.114
Palmitic acid (C16: 0)	23.84	23.93	24.06	0.241	0.819	0.538	0.958
Palmitoleic acid (C16: l ω−7)	4.99	5.42	5.43	0.243	0.374	0.225	0.494
Stearic acid (C18: 0)	6.93	6.50	6.75	0.189	0.309	0.513	0.169
Oleic acid (C18: 1 ω−9)	44.27	44.70	43.41	0.391	0.099	0.145	0.099
Linoleic acid (C18: 2 ω−6)	16.57	16.12	16.51	0.398	0.697	0.909	0.409
γ-Linoleic acid (C18:3 ω−6)	0.20	0.21	0.22	0.007	0.182	0.072	0.824
Linolenic acid (C18:3 ω−3)	0.72	0.72	0.75	0.022	0.496	0.299	0.590
Eicosenoic acid (C20:1 ω−9)	0.56	0.56	0.56	0.015	0.927	0.849	0.742
Arachidonic acid (C20:4 ω−6)	0.62	0.52	0.68	0.107	0.605	0.689	0.366
Eicosapentaenoic acid (EPA; C20:5 ω−3)	0.04	0.06	0.08	0.009	0.050	0.017	0.783
Docosatetraenoic acid (C22:4 ω−6)	0.19	0.16	0.21	0.033	0.573	0.741	0.325
Docosahexaenoic acid (DHA; C22:6 ω−3)	0.00	0.00	0.03	0.020	0.400	0.244	0.493
Saturated fatty acid	31.84	31.57	32.12	0.273	0.393	0.475	0.247
Unsaturated fatty acid	68.16	68.43	67.88	0.273	0.393	0.475	0.247
Monounsaturated fatty acid	49.82	50.64	49.40	0.566	0.322	0.611	0.162
Polyunsaturated fatty acid	18.34	17.79	18.78	0.462	0.550	0.843	0.292

Abbreviation: *T* = overall effects of treatments; *L* = linear effects of increasing inclusion levels of BSFL in diets; *Q* = quadratic effects of increasing inclusion levels of BSFL in diets.

1 Data are least squares means of 5 observations per treatment.

a-c Means within a variable with no common superscript differ significantly (*P* < 0.05).

### Fecal index

Fecal index was not significantly affected by increasing levels of BSFL in the diet. (Table 8). Additional supplementation of BSFL in diets did not affect fecal index incidence compared with a basal diet.

**Table 8:** Effects of increasing inclusion levels of black soldier fly larvae (BSFL) in diets on fecal index of broiler chickens.1.

Items	Inclusion levels of BSFL in diets, %				*P-*value		
	0	1	2	SEM	T	L	Q
Moisture, %	72.3	76.1	74.7	2.22	0.485	0.454	0.351
pH	6.6	6.2	6.2	0.22	0.302	0.227	0.332

Abbreviation: *T* = overall effects of treatments; *L* = linear effects of increasing inclusion levels of BSFL in diets.

1 Data are least squares means of 5 observations per treatment.

## Discussion

### Composition of black solider fly larvae (BSFL)

The BSFL have high nutrients such as protein, fat, and carbohydrate (Herawati and Permata, 2023). In addition, the exoskeleton of BSFL is mainly built of chitin fibers entwined with diverse cuticular protein. Although chitin content in BSFL was not analyzed in our study, it has been reported that the chitin content in BSFL is generally 5.4 % to 9.0 % on DM basis (Caligiani et al., 2018). In addition, chitin has been reported to have negative effects on digestion in broiler chickens (Kim et al., 2021b). However, results of using chitin in animal diets have been inconsistent across previous study (Khempaka et al., 2011). Thus, nutrient digestibility of BSFL may depend on chitin content (Kim et al., 2023). It was expected that the chitin content in BSFL had little negative effect on nutrient digestibility in broiler chickens. The DM concentration of BSFL used in the present study was 96.69 %, similar to the DM concentration (95.7 %) of BSFL meal reported in a previous study (De Marco et al., 2015). The CP (37.9 %) concentration of BSFL was lower than that of BSFL meal (41.1 to 49.0 %; Heita et al., 2023; Makker et al., 2014). This result may be a consequence of changes in fat compositions of BSFL as it was converted to meal. The CF concentration of BSFL used in this study was 29.19 %, similar to the concentrations (18 to 32 %) of BSF reported in previous research (Elangovan et al., 2021). This result may be a consequence of maintaining physiological functions such as energy storage. However, CF concentration of BSFL was higher than that in a previous study (16.71 %; Choi et al., 2013). In addition, high SFAs such as lauric acid (C12:0), myristic acid (C14:0), and palmitic acid (C16:0) in BSFL have been reported (Kierończyk et al., 2023). Similarly, concentrations of SFAs in BSFL used in the present study were high (35.75 % for lauric acid, 5.10 % for myristic acid, and 14.05 % for palmitic acid). These different results may be due to differences in CP, CF, and fatty acid depending on the life cycle of BSFL (Liu et al., 2017).

### Growth performance

In the present study, dietary supplementation of BSFL had no significant effects on BW, BWG, or FI of broiler chickens. The results are consistent with the findings of Choi et al. (2013) and Herawati and Permata (2023). Choi et al. (2013) reported that dietary supplementation of BSFL from 3 % to 6 % had no effect on viability, BW, BWG, FI, or feed conversion ratio (FCR) in broiler chickens. Herawati and Permata (2023) reported that dietary supplementation of 5 %, 10 %, 15 %, and 20 % BSFL had no effect on BW in broiler chickens. These findings suggest that dietary supplementation of full-fat BSFL does not adversely affect growth performance in broiler chickens. Interestingly, our study showed that FE of broiler chickens fed diets containing 1 % BSFL was greater than those fed diets containing 2 % BSFL, although there was no significant difference compared to the control group. This result may be related to differences in nutrient digestibility. According to Spranghers et al. (2018), dietary supplementation of 4 % full-fat black soldier fly improved apparent ileal protein digestibility of weaned piglets, but not at 8 %. In the current study, FI tended to show less 1 % BSFL than 2 % BSFL; however, BW did not differ significantly among treatments. Therefore, the observed differences in FE may be attributed to variations in the digestibility of 1 % and 2 % BSFL.

On the other hand, dietary supplementation of BSFL oil and meal has shown differing effects on growth performance. Schiavone et al. (2017b) reported that replacing 50 % and 100 % of soybean oil with BSFL fat in diets had no effect on BW, ADG, ADFI, and FCR of broiler chickens. Kim et al. (2020a) reported that replacing 50 % and 100 % of soybean oil with BSFL oil in broiler diets had no effect on BW, ADFI, ADG, or FCR. Also, Kim et al. (2022) reported that replacing 50 % of soybean meal with BSFL meal in diets resulted in decreased BW, ADG, and ADFI of broiler chickens. Fruci et al. (2023) also observed that replacing 100 % of soybean meal with BSFL meal in diets negatively affected BW, FI, and FCR of broiler chickens. Overall, growth performance appears to vary depending on the extraction method, supplementation form, and inclusion level of BSFL in broiler diets. In addition, the fat content of BSFL may also contribute to these differences.

### Relative organ weight

The thymus is a key organ of the immune organ systems. It plays a central role in both nonspecific and specific immune responses by supporting the development of crucial immunological components (Fu et al., 2022). Additionally, the thymus is involved in T-lymphocyte differentiation and proliferation (Pearse, 2006). Therefore, thymus development can be used as an indicator of immune function and health status in poultry (Attia et al., 2017; Choi et al., 2021). In our experiment, the relative thymus weight exhibited a quadratic response to increasing dietary concentrations of BSFL. Moreover, the relative thymus weight tended to be greater in the 1 % BSFL compared to both 0 % and 2 % BSFL. These results suggest that dietary supplementation of 1 % BSFL may have a positive effect on immune responses. This finding contrasts with those of Dabbou et al. (2018) and Gariglio et al. (2019). Dabbou et al. (2018) reported that dietary supplementation of defatted BSFL meal at levels of 0 %, 5 %, 10 %, and 15 % had no effect on thymus. In addition, Gariglio et al. (2019) reported that replacing 0 %, 3 %, 6 %, and 9 % of corn gluten meal with defatted meal in diets did not significantly affect the thymus in ducks. The reason for this difference may be due to the use of full-fat BSFL. The BSFL is known to be rich in SFAs, particularly lauric acid (Kierończyk et al., 2023). Pappula et al. (2021) reported that dietary supplementation of lauric acid may increase relative thymus weight of broiler chickens. Lauric acid is a MCFAs with antibacterial properties similar to those of short-chain fatty acids (Pappula et al., 2021). In addition, lauric acid can destruct the cell membrane of Gram-positive bacteria and lipid-coated viruses by physico-chemical processes (Dayrit, 2015). Moreover, lauric acid can increase humoral and cell mediated immune responses (Rout et al., 2016). It has been reported that the antibacterial function of lauric acid in BSFL has positive effects on the thymus (Pappula et al., 2021). Therefore, Therefore, the differences in thymus response observed in our study may be due to varying lauric acid concentrations in the 1 % and 2 % BSFL diets.

### Breast meat quality

Color, fat content, flavor, texture, and price of meat are important factors influencing consumers' purchasing decisions (Brunsø et al., 2005; West et al., 2001). Among these, meat color is a key characteristic of fresh meat (Verbeke et al., 2005), as it serves as an indicator of both spoilage and wholesomeness (Mancini, 2009). Previous studies have reported that consumers prefer a red color in meat (Carpenter et al., 2001). However, our study indicated that increasing concentrations of BSFL in diets linearly decreased a* and b* values of breast meat. Therefore, dietary supplementation of BSFL in broiler diets may influence consumer purchasing behavior by altering meat color. Increasing concentrations of BSFL in diets linearly decreased a* values of breast meat in broiler chickens. This result was different from the study of Schiavone et al. (2019) who reported that dietary supplementation of BSFL defatted meal increased a* values of breast meat in broiler chickens. Adding BSFL to diet may lead to accumulation of insect meal pigments in intramuscular fat, which may affect a* values of breast meat (Schiavone et al*.*, 2019). This result may be associated with differences in accumulation of pigments in intramuscular fat due to the form of BSFL. In addition, this study used BSFL having a thick red or brown color. Therefore, BSFL color affected a* values of breast meat in broiler chickens. On the other hand, increasing concentrations of BSFL in diets linearly decreased b* values of breast meat in our experiment. This result was similar to findings of Schiavone et al. (2019) who reported that increasing concentrations of dietary BSFL defatted meal linearly decreased b* values of breast meat in broiler chickens. To meet energy and nutrient requirements, dietary corn gluten meal concentrations were found to be decreased when inclusion levels of BSFL in diets were increased. In general, corn gluten meal is used as a dietary xanthophyll with yellow pigments (Livingston et al., 1969). In our experiment, two additional diets were formulated with increasing concentrations of BSFL in diets, which decreased corn gluten meal concentrations in diets. The decrease of corn gluten meal concentrations in diets may have decreased b* values of breast meat in broiler chickens. This study showed that increasing BSFL supplementation in diets had a quadratic association with TBARS of breast meat in broiler chickens. Furthermore, birds fed diets containing 2 % BSFL had less TBARS values than those fed diets containing 1 % BSFL. This result was similar to findings of previous experiments. Murawska et al. (2021) reported that feeding diets with supplementing BSFL meal to broiler chickens increased TBARS values. The reason for this result may be associated with lower polyunsaturated fatty acids (PUFAs) in meat, which would be more susceptible to oxidation (Murawska et al., 2021). However, our study did not show a difference in PUFAs concentration among breast meat samples of broiler chickens (Table 7). In our experiment, BSFL contained a high amount of MCFAs such as lauric acid (35.75 %). The MCFAs have been reported to have antimicrobial in mammals and birds (Zentek et al., 2011). In addition, MCFAs have functions in digestion, absorption, transportation, and metabolism (Bach and Babayan, 1982; Caspary, 1992). For this reason, MCFAs can cause oxidation during energy production processes such as digestion, absorption, transportation, and metabolism (Seaton et al., 1986). Thus, lauric acid concentrations in diets may affect TBARS values of breast meat in broiler chickens.

Melanin is a product of natural polymerization of dioxyphenol derivatives (Tyr type) into a high-molecular compound. It is widely present in various organisms with a wide range of biological effects such as antioxidant activity (Bogolyubova et al., 2024). Ushakova et al. (2019) reported that melanin has at least two different types (water-insoluble complex with chitin and chitin-free water-soluble pigment) in BSFL. In addition, two different types of complexes isolated from BSFL contain impurity lipids. The presence of lipids is known to reduce antioxidant properties of water-soluble melanin (Ushakova et al., 2019). This study showed that the increasing inclusion levels of BSFL in diets had no significant effects on melanin concentrations in breast meat. This result is likely to be a consequence of using full-fat BSFL in broiler diets.

### Liver characteristics

Increasing concentrations of BSFL in diets had no effect on liver color, hemorrhagic score, or fatty liver score in broiler chickens. The BSFL having higher concentrations of SFAs generally can cause accumulation of triglycerides (TG) in the liver, which can increase the development of fatty liver. However, the current study showed that increasing inclusion levels of BSFL in diets had no significant effects on fatty liver incidence. This result was similar to findings of previous experiments. Benzertiha et al. (2019) reported that broiler chickens fed diets containing *Tenebrio molitor* (TM) oil show decreased TG and total cholesterol in the serum. The reason for this result may be associated with the fact that dietary TM oil supplementation can increase PUFAs and decrease SFAs of broiler chickens (Benzertiha et al., 2019). The PUFAs can inhibit hepatic lipogenesis, a process by which acetyl-CoA is converted to TG (Pinchasov and Nir, 1992). However, our study did not show differences in concentrations of PUFAs in breast meat of broiler chickens. Kierończyk et al. (2022) reported that turkeys fed diets containing BSFL oil show decreased TG. The reason for this result may be associated with the possibility that BSFL as an energy source can inhibit fatty liver development with anticholesterolemic properties (Kierończyk et al., 2022).

The MDA and TAC are indicators of oxidative stress and antioxidant activity, reflecting the overall antioxidant status of the body (Chen et al., 2022; Kim et al., 2020b; Valle et al., 2015). In the present study, dietary BSFL supplementation did not affect hepatic MDA levels, suggesting no impact on liver antioxidant capacity in broiler chickens. This finding contrasts with the results of Ognik et al. (2020), who reported that dietary supplementation of 0 %, 5 %, 10 %, and 15 % full-fat BSFL meal tended to increase MDA levels in young turkey. On the other hand, Chen et al. (2022) observed that no significant changes in serum MDA when 25 %, 50 %, 75 %, and 100 % of soybean oil was replaced with BSFL oil in broiler diets. Kim et al. (2020b) also reported that dietary supplementation of BSFL oil had no effect on serum MDA levels of broiler chickens. Gariglio et al. (2019) reported found that replacing 3 %, 6 %, and 9 % of corn gluten meal with BSFL meal in diets had no effect on serum MDA levels of ducks. Furthermore, our study found no differences in TAC related to antioxidant capacity in broiler chickens. This result contrasts with the findings of Kim et al. (2020b), who reported that dietary supplementation of BSFL oil increased TAC levels in serum. One possible explanation for these differing results is the fatty acid composition of BSFL. Previous studies have reported that SFAs exhibit greater oxidative stability than unsaturated fatty acids (UFAs; Cosgrove et al., 1987; Mottram, 1998). Therefore, the high SFAs content in BSFL may have contributed to the lack of significant effects on antioxidant capacity in our study. Additionally, tallow was used as a fat source in diets of this study. Tallow is known to contain a higher proportion of SFAs compared to UFAs, similar to BSFL (Kierończyk et al., 2023). Thus, the similarity in fatty acid profiles between tallow and BSFL may have minimized any additional effects of BSFL supplementation on liver antioxidant capacity.

### Fatty acid concentration in breast meat

We observed that increasing concentrations of BSFL in diets linearly increased myristic acid and EPA concentrations in breast meat of broiler chickens. Moreover, myristic acid concentrations of breast meat were increased in broiler chickens fed diets containing 2 % BSFL. These results were similar to findings of a previous experiment. Kierończyk et al. (2022) reported that increasing inclusion levels of BSFL in diets increased myristic acid and EPA concentrations in turkey breast meat. Consistent with previous studies, these results indicated that changing dietary BSFL concentrations can affect fatty acid compositions of breast meat in broiler chicken (de Souza Vilela et al., 2021; Schiavone et al., 2017b). However, our study revealed no significant differences in fatty acids except for myristic acid and EPA concentrations in breast meat. This result is likely to be a consequence of changed fatty acid compositions of BSFL fat according to rearing substrate and age of BSFL (Schiavone et al., 2017b).

## Conclusions

This study demonstrated that BSFL can be effectively utilized as a feed ingredient for broiler chickens. Dietary supplementation of 1 % BSFL resulted in greater improvements in growth performance, immune organ development, and meat quality compared to 2 % supplementation. No adverse effects on antioxidant capacity were observed following BSFL supplementation. Therefore, supplementation of 1 % BSFL in diets is recommended to enhance both performance and health in broiler chickens. However, data on the amino acid composition of breast meat and antioxidant capacity in the liver remain limited. Thus, further research is warranted to elucidate the effects of BSFL on hepatic antioxidant capacity and the amino acid profile of breast meat in broiler chickens.

## Declaration of competing interest

The authors declare that they have no known competing financial interests or personal relationships that could have appeared to influence the work reported in the present study.
